# Learning Upright Standing on a Multiaxial Balance Board

**DOI:** 10.1371/journal.pone.0142423

**Published:** 2015-11-06

**Authors:** Maria Stella Valle, Antonino Casabona, Carlo Cavallaro, Gabriele Castorina, Matteo Cioni

**Affiliations:** 1 Department of Biomedical and Biotechnological Sciences, University of Catania, Catania, Italy; 2 Gait and Posture Analysis Laboratory, University of Catania, Catania, Italy; 3 Physical Medicine and Rehabilitation Residency Program, University of Catania, Catania, Italy; Ludwig-Maximilian University, GERMANY

## Abstract

Upright stance on a balance board is a skill requiring complex rearrangement of the postural control. Despite the large use of these boards in training the standing posture, a comprehensive analysis of the learning process underlying the control of these devices is lacking. In this paper learning to maintain a stable stance on a multiaxial oscillating board was studied by analyzing performance changes over short and long periods. Healthy participants were asked to keep the board orientation as horizontal as possible for 20 sec, performing two sessions of 8 trials separated by 15-min pause. Memory consolidation was tested one week later. Amplitude and variability of the oscillations around horizontal plane and area and sway path of the board displacement decreased rapidly over the first session. The performance was stable during the second session, and retained after 1 week. A similar behavior was observed in the anterior-posterior and medial-lateral directions for amplitude and variability parameters, with less stable balance in the anterior-posterior direction. Approximate entropy and mean power frequency, assessing temporal dynamics and frequency content of oscillations, changed only in the anterior-posterior direction during the retention test. Overall, the ability to stand on a balance board is rapidly acquired, and retained for long time. The asymmetric stability between anterior-posterior and medial-lateral directions replicates a structure observed in other standing stances, suggesting a possible transfer from previous postural experiences. Conversely, changes in the temporal dynamics and the frequency content could be associated with new postural strategies developed later during memory consolidation.

## Introduction

Maintaining upright posture in humans is a complex task which requires a continuous control by the nervous system to overcome the growth rate of the gravitational torque. To guarantee a safe stance in an environment with a variety of perturbations, the strategy of balance control gradually improves during the life span until an adult pattern emerges [1]. Although the acquired upright stance at the end of the development age meets most of daily life activities, further improvements of postural skills can occur during the adulthood. Humans are able to accomplish reactive responses to transient perturbations [2–6], as well as long term adaptations [7–9], producing very complex postural skills, such as those exhibited, for example, by gymnasts or ballet dancers.

Generally, learning a motor skill requires many repetitions over a period of time during which the discrepancy between the current performance and a reference target decreases (error-based paradigm). Throughout trial-by-trial training, the error progressively reduces, tracing a curve with an early phase marked by a rapid improvement in performance, followed by a phase in which the performance approximates the reference target much more gradually. Once the training finishes, the memory consolidation begins to develop and the new skill can be retained over a long time [7–14]. During the retention period, the memory consolidation is accomplished by a complex process with the interaction of several factors, such as the modality of training, the interference with other learning processes, the amount of sleep, and the attentional demand [15–18].

The error-based learning paradigm has been observed across many experimental studies on motor learning, such as reaching in force fields [11], visuomotor adaptation [12], and grip force adaptation [13]. Despite the widespread interest in studying motor learning, systematic investigations to explore the structure of learning and retention of novel upright postures in adults are very sparse. In some cases, the learning based on trial-by-trial training was tested to study the adaptation of upright standing in relation to perturbation of single sensory channels [3, 7–9] or during the uniaxial rotation of the support surface [14].

In the field of rehabilitation, a large consensus exists on the beneficial effects of training with balance boards on many postural rehabilitation contexts, such as the recovery of sport skills [19], the improving of elderly people stability [20], and the treatment of many postural dysfunctions [21–23]. Although the valuable effects of the use of balance boards are widely recognized, we are unaware of any studies that have investigated short and long time changes in the postural control to keep balance on an oscillating board.

To promote a better understanding of the basic adaptation processes and to provide useful suggestions for the dosing and the timing of postural rehabilitation programs, in this work, the issue of learning and memory retention of a novel upright posture was addressed by exploring the ability of healthy young adults to stand and maintain the balance on a board rotating around a multiaxial central pivot.

Unlike previous similar studies, mostly using specific sensory perturbations or surface support rotations around single axes, with a limited parameterization, we adopted a multiaxial balance board making the interactions between the postural control and the environment more challenging.

To keep a multiaxial balance board horizontal, is necessary to activate all the sensory and motor channels to maximize the upright standing performance. This ecological approach is compromised in simple reactive tasks, such as to respond to single perturbation generated by platform servo- controlled or to master a uniaxial balance board. Instead, the more complex process required to cope with a multiaxial board may stimulate the exploration of new schemas of postural control or the use of memorized models from previous experiences. For example, analyzing the oscillations along the anterior-posterior (AP) and medial-lateral (ML) directions within the same functional context, it will be possible to test if the asymmetric control between the two directions observed during static upright stance (AP direction is more perturbed than ML direction; [24]) is also used to control the multiaxial board motion.

For a suitable exploration of these issues, two types of assessment tools were used: stability-related parameters to quantify the reduction of amplitude and variability of the board fluctuations, and a set of measures to evaluate changes in the structure of postural control.

The structure of postural control can change regardless of the level of stability improvement and the phase of learning process. For example, given a level of performance, the spatial and temporal structures of the fluctuations in the AP and ML directions, as well as their power spectrum profile, could show different behaviors depending on postural conditions [24–27], sensory contributions [28, 29], and kinematic strategies [3, 30, 31]. We investigated learning-related changes of the power spectrum by computing the mean power frequency and the spatial and temporal structures of sway variability by using non-linear tools, such as fractal dimension and approximate entropy. Analyses based on these non-linear techniques are now more frequently being used to provide information about the level of complexity of postural control that is not captured by traditional linear measures of variability [32–35].

In this view, the current research pursued two main goals: first, to characterize the time course of learning and retention in maintaining upright stance on a multiaxial balance board; second, to highlight structural changes of postural control as they could emerge from stability asymmetry between the AP and ML directions or from modifications of sway oscillations with respect to the frequency domain and/or temporal dynamics.

## Materials and Methods

### Participants

The participants in this study were ten healthy male college students (24.8 ± 3.3 years; 173.1 ± 5.1 cm; 76 ± 10.2 kg), without any history of neurological diseases or injuries to the lower extremities, and in absence of vestibular or vision disturbances affecting balance. Participants were unfamiliar with the task, and those who had professional experiences in motor performances, such as skiing, skateboarding, martial arts, gymnastics, and ballet were excluded from the study. Standardized instructions and explanations about the procedures were given to the participants just before starting the test.

### Ethics Statement

Participants signed an informed consent document according to the Declaration of Helsinki and this study was specifically approved by the ethical committee of the University of Catania.

### Apparatus and procedures

A force platform (KISTLER 9286 B, Winterthur, CH; 200 Hz sampling frequency) was used to analyze static quiet stance, while a multiaxial balance board (DOMYOS, DECATHLON, Lille, FR) was employed for the balance training.

**Fig 1:**
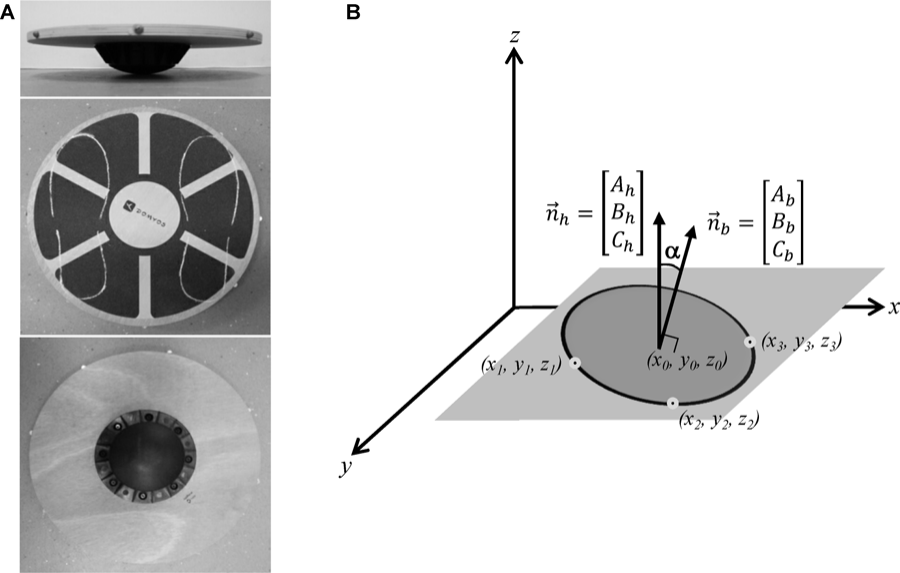
Experimental set up. Balance board (A) used in the experimental procedure and the frame of reference (B) to compute the normal vector for the horizontal (${\vec{n}}_{h}$) and the board (${\vec{n}}_{b}$) plane. The angle α indicates the discrepancy between the two planes. The origin of the reference system is determined by the calibration of the motion capture system. See text for further details.

The balance board used in this study was a wooden plate 40 cm in diameter, 1.5 cm in thickness and 7 cm high, with a rocker base creating an intrinsic multiaxial instability (Fig 1A). Outlines of parallel feet were traced on the board and centered on the center of the board. The two contour lines served as visual reference to ensure that the center of the participant’s feet coincided with the center of the board. Three-dimensional coordinates of eight reflecting markers placed on the edge of the board were captured by eight infrared cameras at the sampling frequency of 200 Hz (SMART-D, BTS, Milan, IT) for the following reconstruction of the board oscillations. The balance board was positioned on the floor, and the base location was marked to avoid board translation.

Prior to the balance training, each participant performed a single 50-sec test of quiet stance on the force platform with the feet parallel and 20 cm spaced at the heels. This configuration was chosen to be consistent with the feet positioning over the balance board.

After a pause of 2 min, participants started the balance training without any preliminary practice to familiarize with the task. Each participant was helped to stand on the board, and, under the supervision of the investigators, he arranged his feet inside the outline borders. Initially, two investigators provided a support on either side of the participant, allowing him to place the board surface parallel to the floor. From this initial position, the trial started when the participant was ready to leave the external support. For each trial, participants were asked to keep the board as horizontal as possible for 20 sec. No instruction was given as to how to keep balance, and the individuals were free to choose their own strategy. During the tests, participants stood barefoot, with their eyes focusing on a mark placed on the wall at a distance of 2.5 m. To prevent falls two investigators were close to the participant during the entire trial.

The experimental protocol included two training sessions (S1 and S2), each of 8 trials, with an inter-session interval of 15 min. Each trial lasted 20 sec with an inter-trial interval of 30 sec. To prevent fatigue, participants were instructed to alert the experimenter if they were feeling fatigue, and the experimenter occasionally asked participants about their fatigue level. Given the large fraction of time for the rest (22 min, including inter-session and inter-trial intervals) with respect to the time for training over the balance board (about 6 min), fatigue was never an issue. A week after the completion of the practice, a retention test of 8 trials was performed to assess the level of memory consolidation of the postural performance. The recording sessions took place in a room at the temperature of 22–24°C, without external noises, and with diffused light.

### Data processing and measurements

Raw signals from the force platform and the markers on the board were captured by the software SmartCapture (BTS, Garbagnate Milanese, MI, IT) and processed offline. All of the data were first interpolated by a cubic spline function to compensate for missing data, and then the signals were low-pass filtered with a zero-lag second-order Butterworth filter with 5-Hz cutoff frequency.

#### Board motion analysis and parameters determination

The motion of the balance board was analyzed by computing the vector orthogonal to the board plane (board normal vector; ${\vec{n}}_{b}$ in Fig 1B). To evaluate the level of stability of the board, the angle α between the normal vector of the board plane and the normal vector of the horizontal plane (that is parallel to the vertical axis; ${\vec{n}}_{h}$ in Fig 1B) was determined. The angle α can be considered the parameter more directly associated with the balance stability: as the postural performance improves the board plane approximates the horizontal plane, and the angle α reduced. The angle α was determined as an absolute value resulting from any combination of pitch and roll board rotations or as separate pitch and roll rotations that are assessments of AP and ML board sways, respectively.

In addition to the angular amplitude measures, the spatial displacement of the board across the horizontal plane and along AP and ML directions was determined. To obtain these measures, the coordinates of the horizontal directional components of the normal vector were computed and the trajectory of the board normal vector over the horizontal plane was traced.

The angle of the board about the vertical axis (yaw board rotation) was obtained by measuring the angle of oscillations of one marker around its initial position before the trial started.

The normal vector computation was performed by using the Cartesian equation of a plane:

Ax+By+Cz=D (1)

where x, y, z are the 3D coordinates of an arbitrary point on the plane measured with respect to the reference system configured for the motion capture apparatus (Fig 1B); A, B, C are the coefficients representing the 3D directions of a normal vector ($\vec{n}$) for the plane: $\vec{n}=A,B,C$; *D* = −*Ax*_0_ − *By*_0_ − *Cz*_0_, where x_0_, y_0,_ z_0_ are the 3D coordinates of the normal vector position.

The angle of the board with respect to the horizontal plane was determined by computing the directional coordinates A, B, C, of the normal vector given three points lying on the plane. To select the three points among the 8 markers applied on the board, the trajectories of each marker was tracked, and the 3 best signals (with less missing values) were chosen.

The directional coordinates for an equation of the plane passing through the points (x_1_, y_1_, z_1_), (x_2_, y_2_, z_2_), and (x_3_, y_3_, z_3_) are given by the following determinants:

$$A=\left|\begin{array}{ccc} 1 & y_{1} & z_{1}\\ 1 & y_{2} & z_{2}\\ 1 & y_{3} & z_{3} \end{array}\right|\;B=\left|\begin{array}{ccc} x_{1} & 1 & z_{1}\\ x_{2} & 1 & z_{2}\\ x_{3} & 1 & z_{3} \end{array}\right|\;C=\left|\begin{array}{ccc} x_{1} & y_{1} & 1\\ x_{2} & y_{2} & 1\\ x_{3} & y_{3} & 1 \end{array}\right|$$ (2)

The direction of the normal vector for the horizontal plane (${\vec{\text{n}}}_{\text{h}}={\text{A}}_{\text{h}},{\text{B}}_{\text{h}},{\text{C}}_{\text{h}}$) was determined by capturing the coordinates of the three points when the board was on the floor without load, and after the checking of the horizontal alignment by means of a bubble level. Instead, the normal vectors for the board planes that follow one another over the 20-sec trial (${\vec{n}}_{b}=A_{b},B_{b},C_{b}$), were computed by the coordinates captured bin-by-bin. To simplify the computation of the board angle, the normal vectors ${\vec{\text{n}}}_{h}$ and ${\vec{\text{n}}}_{\text{b}}$ were normalized for their lengths obtaining the unit normal vectors ${\hat{n}}_{h}$ and ${\hat{n}}_{b}$. Thus, for each data sampling, the current angle (α) of the board with respect to the horizontal plane was computed as follows:

$$\alpha ({\hat{n}}_{h},{\hat{n}}_{b})=arc\;cos|A_{h}∙A_{b}+B_{h}∙B_{b}+C_{h}∙C_{b}|$$ (3)

Angle amplitude measures were converted from radiant to degree.

The analysis of the spatial displacement of the board normal vector over the horizontal plane by using the two directional components A and B, was the basis to determine the following parameters: total area covered by the board normal vector computed as the 95% confidence ellipse (see [36]); shape of the ellipse obtained by computing the minor to major axis ratio; total length travelled by the board normal vector (sway path) across the horizontal plane and along AP and ML directions; root mean square (RMS) calculated for each time series associated with AP and ML directions.

In addition to linear measurements of postural stability, we computed the Approximate Entropy (ApEn) and the Fractal Dimension (FD) as non-linear measures to explore the temporal dynamics of the signals along AP and ML directions, and the structure of the planar trajectory tracked by the board normal vector.

Approximate Entropy is a probability statistic that calculates the likelihood that a template pattern repeats over time. It is frequently being used to estimate the variation of regularity in time series associated with physiological signals [37, 38], including postural data [7, 32–35, 39, 40]. According to preliminary tests and the protocols established in former studies, input parameters for the ApEn calculation were the length of the time series (4000 points), a pattern length of 2 data points, a tolerance window normalized to 0.2 times the standard deviation of individual time series, and a lag value of 10. For a detailed explanation of the mathematical procedures to calculate the ApEn see Pincus [37].The outcome of the ApEn calculation ranges between 0 and 2, with a decreasing of regularity in the signal as the value moves toward the highest edge. We obtained a single ApEn value for each time series associated with AP and ML directions.

The Fractal Dimension (FD) was computed using the algorithm described by Prieto et al., [36], who quantified the level of geometrical complexity of a planar trajectory as follows:

FD=log(N)/log(N∙d/Swaypath) (4)

where N is the number of data points (N = 4000); *d* = (2*a* ⋅ 2*b*)^1/2^ where *a* and *b* are the major and the minor axes of the 95% confidence ellipse, respectively; *Sway path* is the total length travelled by the board normal vector. Two-dimensional FD ranges from 0 to 2, with the value increasing when the trajectory is more complex.

To explore the frequency domain of the postural sway, the Power Spectral Density (PSD) was performed on the unfiltered AP and ML time series using the multitaper estimation method, as suggested by Prieto et al., [36]. This analysis was carried out sampling the frequency spectrum in bins of 0.025 Hz. The first bin was removed and, since no discernable spectral peaks were visible above 1.5 Hz, we collected signals within a range of frequencies between 0.025 and 1.5 Hz. The total area of the PSD and the Mean Power Frequency (MPF) were calculated for the power output in AP and ML directions. MPF represents the mean frequency contained within a power spectrum and was determined as follow:

MPF=∑f∙P(f)/∑P(f) (5)

where *f* represents frequencies in the signal and *P* is the amplitude of the PSD at each frequency.

All the computations were performed using a customized MatLab R2012a code (Mathworks, Natick, MA, USA).

#### Parameters determination for the static stance

The center of pressure (COP) of static stance was computed from forces and torques measured by the force platform and elaborated by the software Sway (BTS Garbagnate Milanese MI, IT). To identify similar postural patterns between quiet stance and stance on the oscillating board, we carried out measurements of sway path, RMS, ApEn, and MPF for the COP along AP and ML directions. The determination of these parameters was performed following the processes described in the previous section, replacing the unit normal vector trajectory with the COP trajectory.

#### Statistical analysis

For each trial the mean and the variability, expressed as Standard Deviation (SD), of each parameter were determined participant by participant, and grand averages over the 10 participants were computed.

Statistically significant changes across sessions and trials were estimated using a two way Analysis of Variance (ANOVA) with repeated measures, considering sessions and trials as within subject factors. Statistical differences between the AP and ML directions were evaluated by a three way ANOVA with repeated measures, with sessions, trials and directions as within subject factors. The critical value of F was adjusted applying Greenhouse-Geisser correction, which produces a p-value more conservative. This procedure corrects the repeated-measures ANOVA with respect to a possible violation of the sphericity assumption, that is, the variance of the differences among all combinations of independent variables must be equal. Post hoc tests with Bonferroni correction for multiple comparisons were conducted to identify local significant differences between sessions or trials. The level of statistical significance was set to P < 0.05.

The sample size, to detect differences in stability and structural parameters over the sessions, was determined *a priori* based on preliminary data acquired from 5 participants. Power calculations for within-subjects designs were performed using G-power (version 3.1.9.2; [41]) by taking specification as in Cohen [42] and giving partial eta squared (η^2^_p_) as input. Power analysis revealed that a sample size of 7 was required to achieve a power of 0.95 for 10 out of 12 parameters, while only for the sway path (2D path and along AP and ML directions) a sample size of 10 was required to achieve a power of 0.85. On this basis, we deemed that 10 participants was a sufficient quantity for the results to be meaningful.

The magnitude of significant differences (effect size) for the multivariate within-subjects ANOVA statistics was estimated by using η^2^_p_, which describes the percentage of variance of the dependent variable attributed to the independent variables of interest. For the post-hoc paired tests, the effect size was determined by using Hedges’ g_av_, which represents a correction for bias of Cohen's d_av_ (the standardized mean differences based on the *average* standard deviation of both repeated measures). The effect size computations was based on the recommendation reported by Lakens [43].

Statistical analysis was performed using SYSTAT, version 11 (Systat Inc., Evanston, IL, USA) and Matlab version R2012a (Mathworks Inc, Natick, MA, USA).

## Results

**Fig 2:**
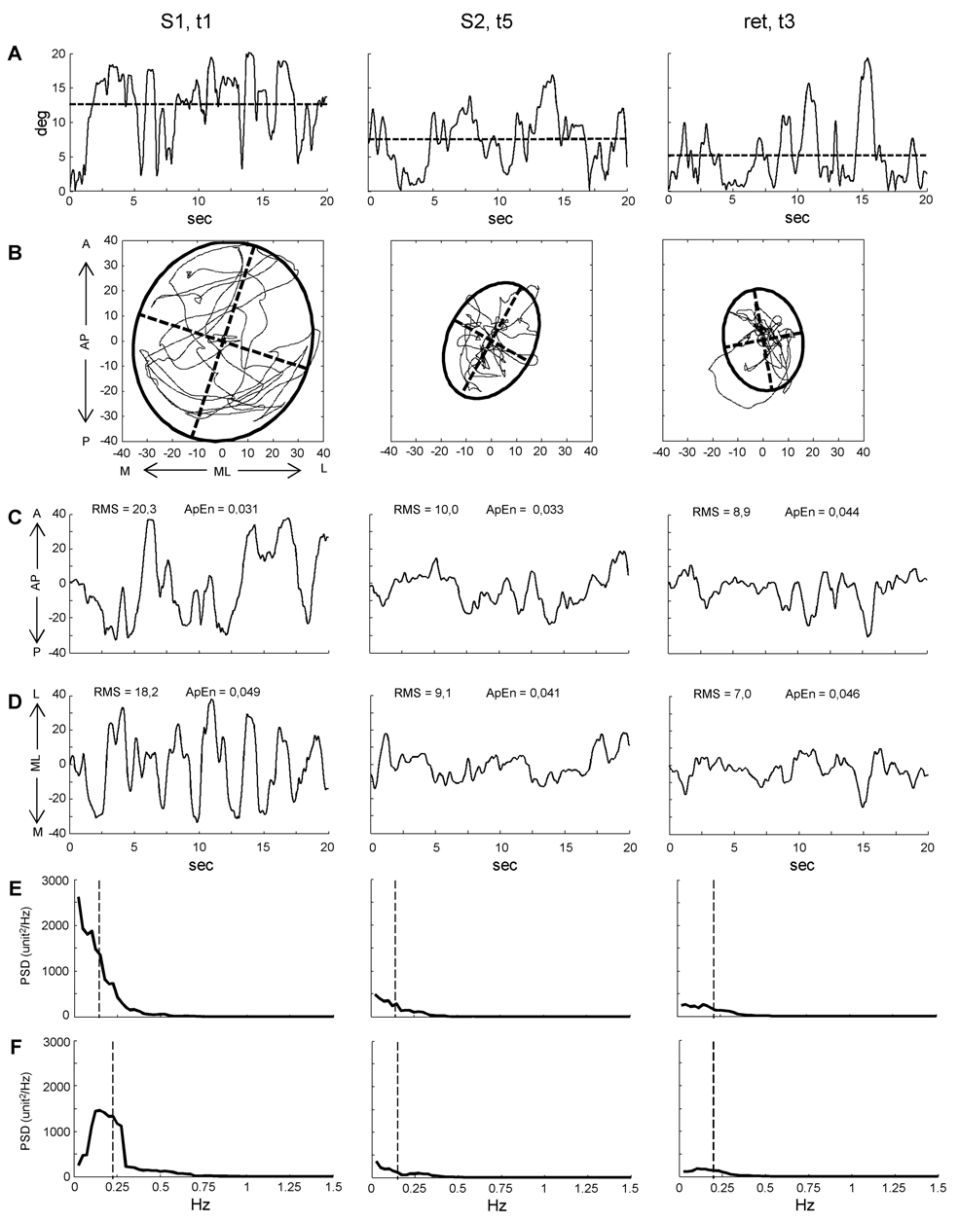
Representative example of the postural outcome. The plots illustrate the effect of practice on the balance board motion exhibited by one participant during three trials (t1, t5, t3) performed across three sessions (S1, S2, retention). (A) Amplitude of angular oscillations of the board around the horizontal plane regardless of the direction. Dashed lines indicate the mean values. (B) Area covered by the unit normal vector expressed as 95% confidence ellipse. (C and D) Amplitude of oscillations of the unit normal vector along Antero-Posterior (AP) and Medial-Lateral (ML) directions. (E and F) Power Spectral Density analysis performed in AP and ML directions. Dashed lines indicate the Mean Power Frequency. The axes values reported in B and in vertical axes of C and D derive from measurements of the space as unit vector.

A representative example of data obtained from one participant is illustrated in the Fig 2. The board oscillations approximated the horizontal plane (0 degree) as the training evolved from the trial 1 (t1) of the S1 to the trial 5 (t5) of the S2. The acquired performance was maintained in the retention session (Fig 2A). The training also had a similar effect on the spatial motion of the board across the horizontal plane, with reduction and final consolidation for the area covered by the trajectory of the board normal vector (Fig 2B), and for the oscillations amplitude in the AP (Fig 2C) and ML (Fig 2D) directions. The amplitude and the variability (RMS) of the fluctuations were higher along the AP than ML direction across the three trials. However, when the variability was measured as ApEn the two directions diverged in S1 and S2, but showed similar values during the retention session. The profiles of the PSD for the AP (Fig 2E) and ML (Fig 2F) directions exhibited a reduction of the amplitude of the frequency at the lower band, with more similar values of MPF (vertical dashed lines) between the two directions in the retention test.

Most of the changes reported in this example are replicated in the results of the statistical analyses performed in the entire sample and described in the following sections.

### Changes in amplitude of the board oscillations

**Fig 3:**
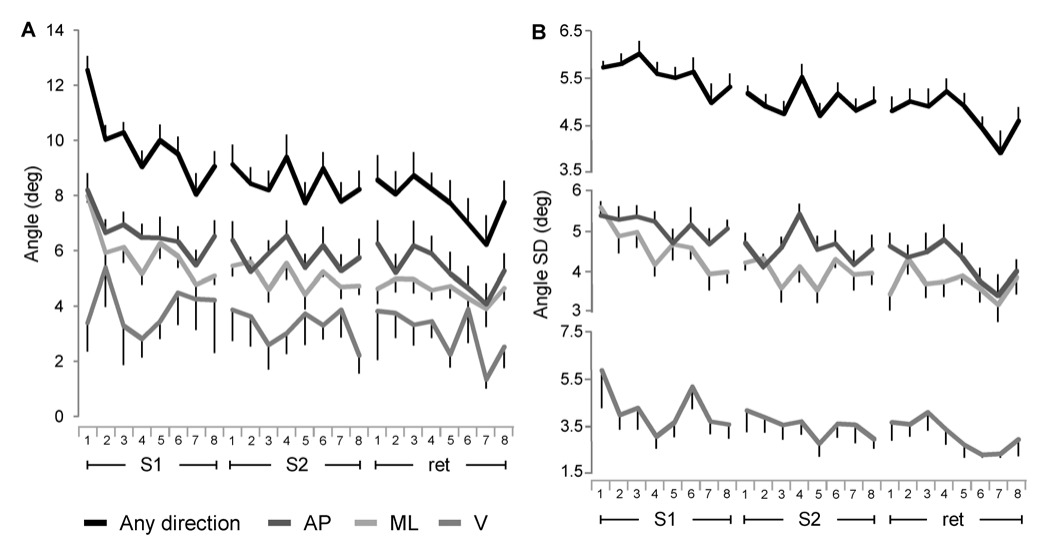
Changes in angular oscillation of the board across the time course of learning. Amplitude (A) and variability (B) of angular oscillation of the board around the horizontal plane for any direction (first plot from the top), for AP and ML directions (second and third plot from the top) and around the vertical axis (most bottom plot). The error bars represent the standard errors. Abbreviations as in Fig 2.

The ability to maintain the balance board aligned with the horizontal plane improved progressively across the trials of S1 of training, reaching a stable level during S2. After one week, the level of performance was preserved, with a further improvement in the last trials (Fig 3A, 1st row from top). Analyzing changes in the angular amplitude of oscillations around the horizontal plane, there were sessions and trials main effects and a significant interaction for the two factors (Table 1, parameter 1). The post hoc details revealed significant differences between S1 and the following two sessions, but there were no differences between S2 and the retention session. The angular discrepancy between horizontal plane and the board plane changed significantly comparing the first trial (12.5±0.5°SE) with each of the following trials (P < 0.01). At the end of the S1 the angle difference reached a value (9.1±0.6°SE) that exhibited no significant changes across the S2 and the first 5 trials of the retention session. The performance started to improve again at trials 6 and 7 of the retention session with respect to the trial 1 of the same session (P < 0.05), reaching the minimum angle error at trial 7 (6.2±1.1°SE).

**Table 1:** Summary of statistical analysis for any direction board motion.

Parameters		Sessions	Trials	Sessions X Trials	Sessions post hoc		
		df: 2, 18	df: 7, 63	df: 14, 126	S1/S2	S1/ret	S2/ret
**1. Horizontal rotation**	F	11.232	6.6587	2.488			
	P	**0.0007**	**<0.0001**	**0.0044**	**0.0088**	**0.0112**	NS
	η^2^_p_	**0.56**	**0.43**	**0.21**	0.83*	1.11*	
**2. Vertical rotation**	F	1.0540	0.9487	0.6313			
	P	0.3691	0.4762	0.8347	NS	NS	NS
**3. SD Horizontal rotation**	F	46.0791	2.6465	1.1137			
	P	**<0.0001**	**0.0183**	0.3523	**0.0001**	**0.0001**	**0.0241**
	η^2^_p_	**0.84**	**0.23**	**0.11**	1.03*	1.59*	0.41*
**4. SD Vertical rotation**	F	2.4009	1.8500	1.4597			
	P	0.1191	0.0932	0.1359	NS	NS	NS
**5. Displacement Area**	F	62.0432	9.1037	4.418			
	P	**<0.0001**	**<0.0001**	**<0.0001**	**0.0001**	**<0.0001**	**0.0016**
	η^2^_p_	**0.87**	**0.50**	**0.33**	1.01*	1.86*	0.69*
**6. Sway path**	F	11.6167	6.7895	3.8143			
	P	**0.0006**	**<0.0001**	**<0.0001**	**0.0011**	**0.0113**	NS
	η^2^_p_	**0.56**	**0.43**	**0.30**	1.30*	1.12*	
**7. Ellipse Axes ratio**	F	0.0044	0.6413	0.7964			
	P	0.9956	0.7201	0.6715	NS	NS	NS
**8. FD**	F	9.8086	1.2562	0.5177			
	P	**0.0013**	0.2866	0.9192	NS	**0.0209**	**0.0326**
	η^2^_p_	**0.52**				0.72*	0.67*

NS, not significant; S1, session 1; S2, session 2; ret, retention session. In the shadow cells are reported the effect sizes expressed as partial eta squared (η^2^_p_) for the ANOVA factors, and as Hedges g_av_ (*) for the sessions comparison. Significant values and their effect sizes are indicated in bold.

Similar significant differences, with some local rate variations, were observed for the board angle fluctuations along the AP direction (Fig 3A, 2nd row from top) and along the ML direction (Fig 3A, 3rd row from top). In this case, significant differences were also found between the two directions, with AP angular values larger than ML across the three sessions (Table 2, parameter 1). No significant differences were found for the angle of board rotation around the vertical axis (Fig 3A, 4th row from top; Table 1, parameter 2).

**Table 2:** Summary of statistical analysis for signals in AP and ML directions.

Parameters		Direction	Sessions	Trials	Direction X Sessions	Sessions post hoc		
		df: 1, 9	df: 2, 18	df: 7, 63	df: 2, 18	S1/S2	S1/ret	S2/ret
**1. AP/ML Rotation**	F	5.8976	11.5628	6.7671	0.0081			
	P	**0.0381**	**0.0006**	**<0.0001**	0.9919	**0.0095**	**0.0094**	NS
	η^2^_p_	**0.40**	**0.56**	**0.43**		0.81*	1.11*	
**2. SD AP/ML Rotation**	F	6.7083	48.2929	3.3391	0.1091			
	P	**0.0292**	**<0.0001**	**0.0043**	0.8973	**0.0001**	**0.0001**	**0.0102**
	η^2^_p_	**0.43**	**0.84**	**0.27**		1.00*	1.65*	0.58*
**3. AP/ML Sway path**	F	0.3253	12.1690	6.5222	3.6098			
	P	0.5824	**0.0005**	**<0.0001**	**0.0481**	**0.0010**	**0.0091**	NS
	η^2^_p_		**0.57**	**0.42**	**0.29**	1.30*	1.14*	
**4. AP/ML RMS**	F	9.5417	18.3059	5.9465	0.0986			
	P	**0.0130**	**<0.0001**	**<0.0001**	0.9066	**0.0020**	**0.0021**	NS
	η^2^_p_	**0.51**	**0.67**	**0.40**		0.88*	1.26*	
**5. AP/ML ApEn**	F	1.7654	16.8407	1.5114	3.8621			
	P	0.2206	**0.0001**	0.1823	**0.0428**	NS	**0.0170**	**0.0017**
	η^2^_p_		**0.65**	**0.30**			0.78*	0.89*
**6. AP/ML PSD**	F	7.4196	22.3953	8.4141	0.1023			
	P	**0.0235**	**<0.0001**	**<0.0001**	0.9033	**0.0003**	**0.0008**	NS
	η^2^_p_	**0.45**	**0.71**	**0.48**		0.54*	0.83*	
**7. AP/ML MPF**	F	5.5134	12.7636	2.1052	3.9031			
	P	**0.0468**	**0.0005**	0.0578	**0.0416**	NS	**0.0426**	**0.0023**
	η^2^_p_	**0.38**	**0.59**		**0.30**		0.43*	0.60*

NS, not significant; S1, session 1; S2, session 2; ret, retention session. In the shadow cells are reported the effect sizes expressed as partial eta squared (η^2^_p_) for the ANOVA factors, and as Hedges g_av_ (*) for the sessions comparisons. Significant values and their effect sizes are indicated in bold.

A parallel behavior was observed for the variability of angular amplitude, expressed as SD, for the board oscillations in any direction of the horizontal plane (Fig 3B, 1st row from the top) and in AP and ML directions (Fig 3B, 2nd and 3rd row from the top). Although the variability parameters exhibited smaller rate of reduction over the S1 with respect to the reduction of the angle amplitudes, there were significant differences between sessions and trials for the SD of the horizontal angle rotation and for all the paired comparisons between the sessions (Table 1, parameter 3). The variability along AP direction showed also in this case significant larger values than the variability observed along ML axis (Table 2, parameter 2). No significant differences were again found for the variability of the angle of rotation around the vertical axis (Fig 3B, 4th row from top; Table 1, parameter 4).

Among the parameters which exhibited significant differences, the sessions explained most of the variance with bigger effect sizes than the other factors. The pairwise comparison between the sessions showed the size of the effect of S1 vs S2 and S1 vs retention stronger than the effect size of S2 vs retention.

### Spatio-temporal analysis of the board displacement

**Fig 4:**
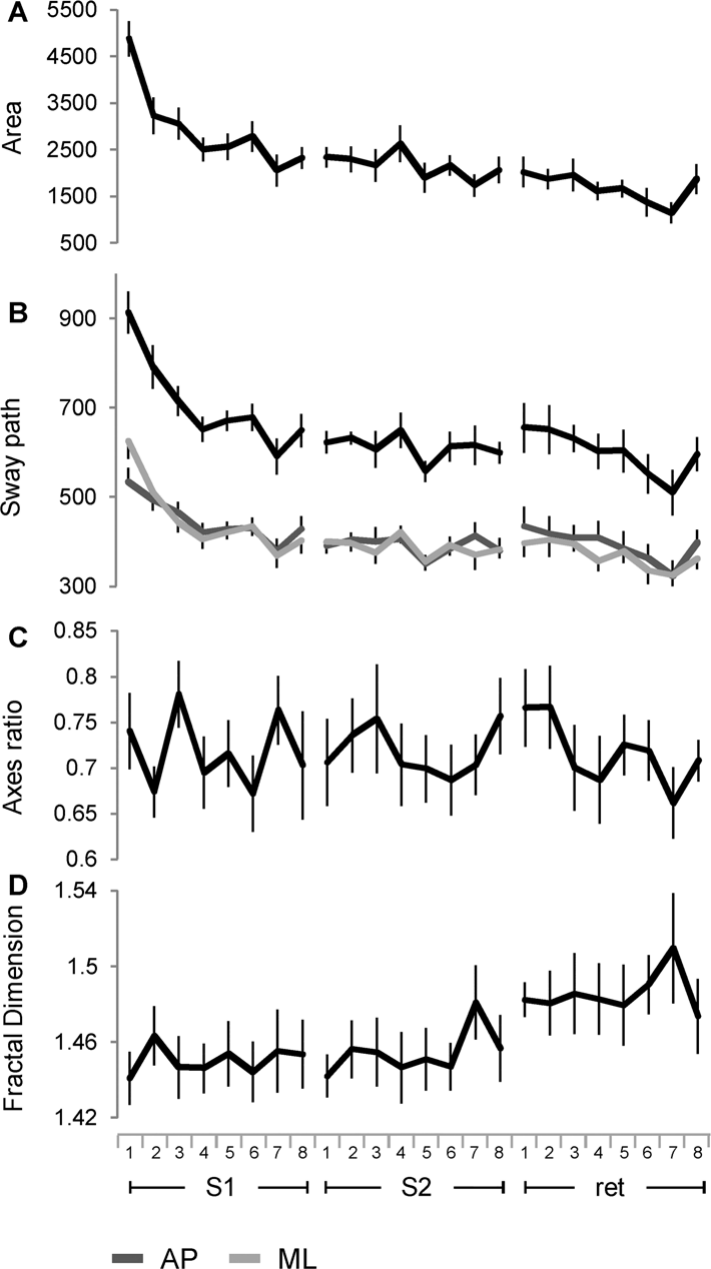
Changes in horizontal displacement of the board across the time course of learning. Area (A), sway path (B), 95% confidence ellipse axes ratio (C) and fractal dimension (D) computed from the trajectory of the unit normal vector. Symbols, units and abbreviations as in Figs 2 and 3.

To characterize the motion of the balance board over the space of the horizontal plane and to verify the level of sensitivity of spatial parameters with respect to the learning process, we considered the displacement on the horizontal plane of the board normal vector. The changes of the total area of the board displacements showed large significant differences for the main effect of sessions, trials, and their interaction, as well as for the paired post hoc comparison among the three sessions (Fig 4A; Table 1, parameter 5). Except for no significant change in the paired comparison between S2 and the retention session, a similar trend was exhibited by the total length of the sway path covered by the board motion (Fig 4B, 1st row from top; Table 1, parameter 6). The inter-trial differences for the area and the sway path are comparable to those observed for the horizontal angular rotation. The variations of sway path in relation to AP and ML axes were significant with main effect of session, trials and the interaction between direction and session (during retention session the two axes showed more divergence than in the previous sessions), but no significant difference was observed between the two directions (Fig 4B, 2nd and 3rd row from top; Table 2, parameter 3).

The determination of the area by the 95% confidence ellipse gives information also on the shape taken by the surface covering the trajectory of the board normal vector. This quantification may be captured by the minor to major ellipse axis ratio: as this value approximate 1 the shape of the ellipse turns into a circle. The ellipse axes lengths ratio was unchanged across sessions and trials indicating an invariant shape of the ellipse across the learning and consolidations sessions (Fig 4C; Table 1, parameter 7).

The level of complexity of the spatial trajectory traced by the balance board was assessed by computing the FD (Fig 4D). There was a main effect of the sessions, with the retention session showing significant higher level of complexity with respect to the other two sessions (Table 1, parameter 8). Local significant differences occurred between the trial 7 of the S2 and most of the previous trials (P < 0.05).

The values of effect sizes for the spatial parameters exhibited the same trend of angular amplitude, with the sessions explaining most of the variance. S1 vs S2 and S1 vs retention showed again the highest effect sizes among post hoc comparisons, except of the FD, which showed valuable effect sizes only between retention and each of the two training sessions.

**Fig 5:**
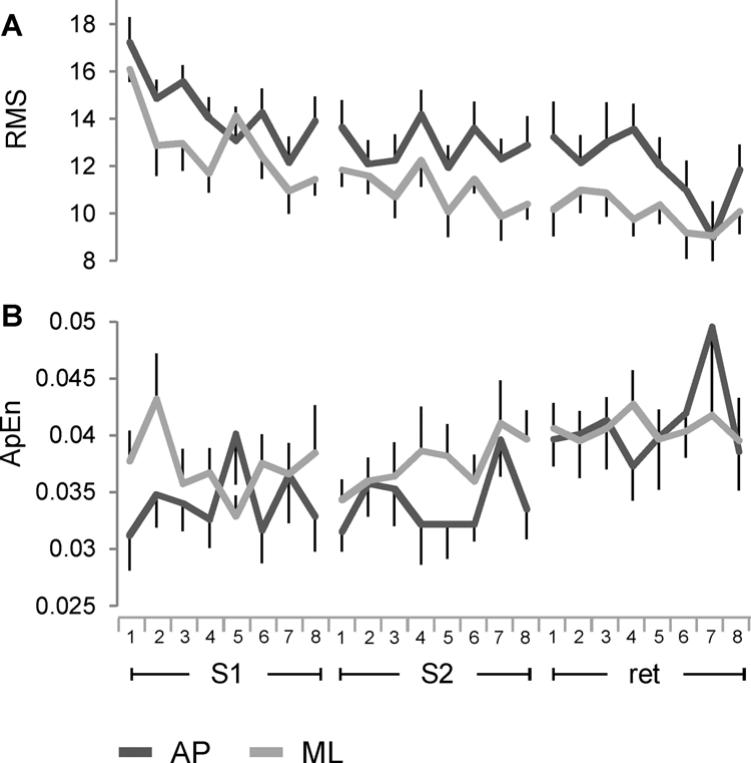
Changes in variability along AP and ML directions across the time course of learning. Root Mean Square (A) and Approximate entropy (B) computed from the time series of the unit normal vector along AP and ML directions. Symbols, units in A and abbreviations as in Figs 2 and 3.

Temporal variations in the board normal vector oscillations along AP and ML axes were estimated by linear (RMS; Fig 5A) and non-linear (ApEn; Fig 5B) parameters. RMS exhibited significant differences for directions, sessions, and trials with the largest variance explained by sessions. The values in AP direction were higher than the values in ML direction across the sessions, and there was no significant interaction between directions and sessions. Post hoc analysis indicates significant changes between S1 and S2 and between S1 and the retention session (Table 2, parameter 4). The ApEn showed a main effect for the sessions and a significant interaction between the directions and the sessions (Table 2, parameter 5). There were significant differences between each of training session and the retention, with valuable effect sizes. No significant difference was exhibited between S1 and S2. The ApEn values in AP direction were lower than ML direction during S1 and S2, but the level of regularity of the signal in the two directions converged during the retention session.

### Frequency domain analysis of the board oscillations

**Fig 6:**
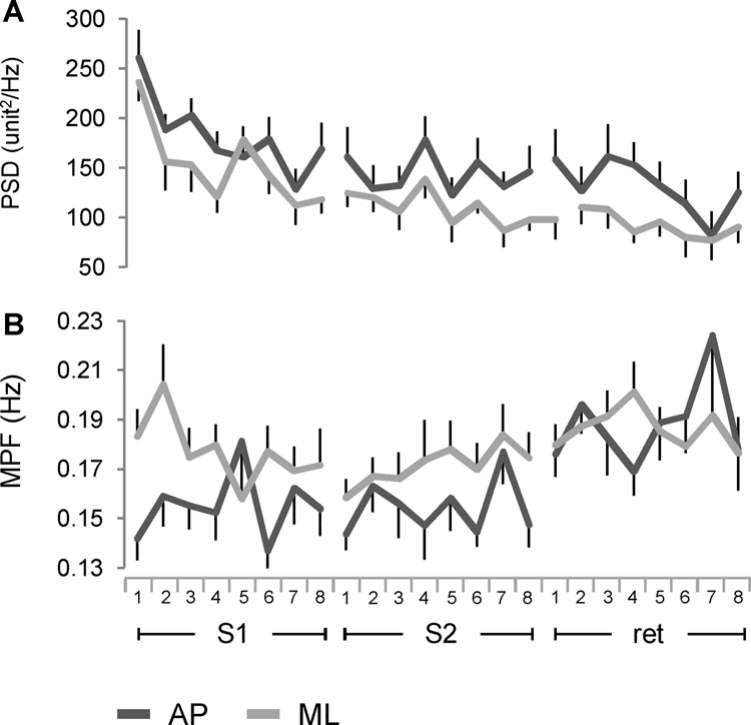
Changes in frequency domain across the time course of learning. Power Density Spectrum (A) and Mean Power frequency (B) computed from the time series of the unit normal vector along AP and ML directions. Symbols unit in A and abbreviations as in Figs 2 and 3.

The results of the PSD analysis for AP and ML board oscillations are summarized in Fig 6. The PSD resembled the behavior of the angular and spatial parameters, with a significant reduction for the direction, the sessions, and the trials, but no significant differences there were for the interaction between directions and sessions. (Fig 6A; Table 2, parameter 6). Sessions and directions were significantly different for the MPF and there was a significant interaction between directions and sessions. During S1 and S2, the values in the ML were higher than in AP direction, but in the retention session the MPF increased only for the AP direction converging to the level of the ML direction. Only the comparison between retention and the training sessions showed significant differences, with the size of the effect of S2 vs retention larger than S1 vs retention (Fig 6B; Table 2, parameter 7).

### Analysis of the COP during the static stance

**Fig 7:**
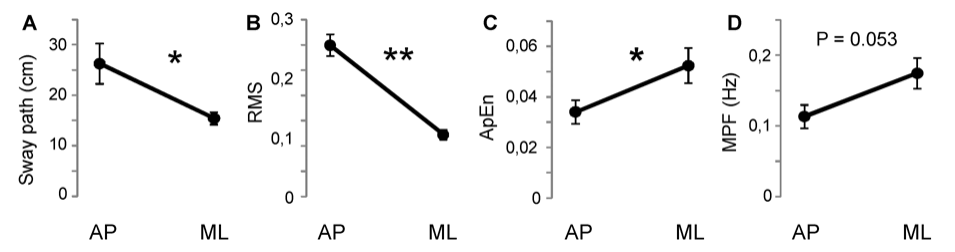
Changes during quiet bipedal stance. Sway path (A), Root Mean Square (B), Approximate entropy (C) and Mean Power frequency (D) between AP and ML direction. The data were obtained by the computation of the center of pressure. * P < 0.05; ** P < 0.001. Other symbols and abbreviations as in Fig 3.

To compare changes in AP and ML directions during quiet static stance, a set of parameters was computed from COP data (Fig 7). Sway path and RMS were higher in the AP than in ML direction (Fig 7A and 7B), while a reversed relation was observed for ApEn and MPF (Fig 7C and 7D). The differences were statistically significant for all the parameters, except for the MPF that showed a marginal level of significance (Sway path: t = 3.07, P = 0.015; RMS: t = 8.04; P < 0.001; ApEn: t = 2.56, P = 0.033; MPF: t = 2.26, P = 0.053).

## Discussion

The data reported in this study show that learning to stand up on a multiaxial balance board is accomplished by reducing both amplitude and variability of the board oscillation, within a short time range of practice. Over the training and retention sessions, the anterior-posterior direction was more instable than the medial-lateral direction. During the retention test, the performance preserved the level of stability acquired, and structural adaptations in the temporal and frequency domain parameters specifically appeared in the oscillations along AP direction.

### Learning a challenging upright standing posture

The postural adaptations described in this paper are in line with the error-based learning paradigm reported for other motor tasks [11–13]. The angular discrepancy between the board and the horizontal plane decreased fast within the first few trials followed by a more gradual phase in which performance gains increase much more slowly. A parallel reducing of the variability was also observed, and all the performance parameters were stable after one week pause.

To the best of the authors’ knowledge, few works approached the study of learning and adaptation of upstanding posture by comparing trial-by-trial changes during short and long time windows. Using an experimental design similar to that adopted in this work, Shea et al., [14] found a reduction of sway variability on a board oscillating along ML direction. Short and long-term learning was also observed when the adaptation was associated with single vestibular [9] or proprioceptive perturbations [7, 8]. To compare our results with the above-mentioned studies, the type of elaboration and the level of task complexity have to be taken into account.

When a single sensory channel is perturbed, the main adaptation accomplished by the nervous system is to suppress the extraneous information and to reweight that specific channel [44]. In our task each sensory pathway provided reliable information on the current mechanical status, thus, rather than a sensory reweighting, the challenge was to integrate these signals to assist the formation of a novel postural skill.

The complexity of the postural control increases as the number of sway directions increases. Therefore, when the direction of board oscillations was limited to a single axis [7, 14], the system faced with less degrees of freedom and more predictable perturbations with respect to those exhibited by the multiaxial board used in our work.

Considering the specificity of the postural skill of these experiments and the data from studies exploring different motor tasks [7–14], our results support the idea that a general scheme in the timing of motor learning and consolidation is maintained regardless of sensory information, task complexity, body segments involved, and the mode of interaction with the environment.

When a task is achieved by multiple segments and in conditions of high variability, the error-based paradigm will not be the only mode to accomplish the learning procedure [10]. In fact, parallel processes can be implemented in order to reduce the energy expenditure and to optimize the combination of the diverse components [45; 46]. For example, in a pointing movement a given level of accuracy can be obtained by using many hand trajectories and patterns of segments coordination [45]. Thus, a goal of the motor learning is also to select behaviors to gain the lowest error with the lowest effort. The area and the sway path of the board motion may provide an estimation of the mechanical energy employed in the control of board oscillations. In fact, there are several cases where changes in mechanical work are associated with similar angular rotations. For example, when the board rotates around the vertical axis the range of angular rotation could be unchanged, although the work executed increases as the path and the area of the board motion changes. Therefore, area and sway path reductions observed in this paper suggest that the balance training on a multiaxial board not only increases the accuracy of the performance, but also minimizes the energy expenditure.

### Structural changes during the learning of the postural performance

This study provides some insights into the nature of underlying adaptation processes occurring during the practice of a novel standing posture. In particular, the differences in stability observed between AP and ML directions, and the changes in ApEn and MPF occurred during retention session suggest the existence of specific schemas of postural control.

#### Anisotropic stability between AP and ML directions

The postural performance showed a parallel improving in AP and ML directions, but several spatial and temporal features of the board fluctuations followed an asymmetric distribution between the two directions: angular rotation amplitude, motion variability and total PSD were higher in AP than in ML direction.

Generally, the discrepancy of stability between AP and ML directions depends on the combination of joints involved in the balance task. Based upon the model proposed for the first time by Nashner and McCollum [47] and supported by several other authors [4, 24], humans use two discrete joint strategies: the ankle strategy where the body behaves as a single segment-inverted pendulum by producing muscular torques around the ankle, and the hip strategy, with the body moves as a double-segment inverted pendulum by rotations of hip and ankle in opposite directions. Given the ankle anatomy, the ankle strategy constrains the body sway mainly along the sagittal plane increasing the instability in the AP direction, while, as the hip strategy is used, the multiaxial rotation at hip joint may perturb the body along both the directions. However, as the posture passes from quiet, side-by-side standing, to more perturbed conditions, the ankle and hip strategies may be concurrently activated, producing a less discrete postural behavior [2, 5, 6, 25, 48, 49]. Some authors deemed that ankle and hip strategies are inadequate in representing a general model of upright standing, suggesting that the control variable for the posture adaptations is the level of coordination dynamics between joints and not the motion of a single joint [50, 51]. Moreover, other joints, such as the knee, may show a significant contribution for both quiet and perturbed stance [51–53].

In line with this view, the multiaxial perturbation and the number of body segments used to perform our task would suggest that a discrete selection of ankle or hip strategy is inadequate to explain the anisotropic behavior between AP and ML directions. Rather, a more plausible explanation may be achieved by considering patterns of interjoint coordination and muscle synergies able to constrain most of the body oscillations along the AP axis.

During the board fluctuations along AP direction, the body weight is distributed between the heel and the toe, and the board motion is controlled by the activity modulation of flexor and extensor muscles of the ankle, knee, and hip joints. Instead, when the board oscillates along ML direction the body alternatively loads one foot while unload the other. In this case, ankle and knee rotations around anterior-posterior axis are limited by their anatomical constrains, and the load/unload behavior mainly depends on the muscle modulation of the hip abductor/adductor activity. Such a synergy for the control of hip motion was proposed by Winter et al., [24] to explain differences between AP and ML balance during quiet standing with different feet configurations. Flexor/extensor and abduction/adduction modulations of ankle, knee and hip muscles were found also during postural adjustments in response to surface translation along different perturbation directions [6, 49]. We acknowledge that our data are inadequate to support interpretation based on muscle dynamics since no electromiographic recording was performed. However, the intrinsic stability of ankle and knee joints in ML direction and the control of abduction/adduction synergy to limit ML oscillations at hip joint may be a parsimonious functional schema to explain the predominant fluctuations along AP direction observed in this study.

Although this interpretation suggests an intrinsic contribution of joint mechanical constrains to the anisotropic stability observed between the two orthogonal directions, this behavior may also arise from memorized strategies produced during previous postural experiences and adapted to the novel task performed in the current study. In fact, large body oscillations in the AP direction are common in daily living activities, such as taking a step or standing up from the chair. The results reported for the static posture performed by our participants confirm the data from other studies [24, 36, 54] and support the presence of a comparable structure between quiet standing and the stance on the balance board. Moreover, the AP-ML discrepancy was also replicated during postural perturbations associated with stability of the support surface [2, 4, 5, 25, 26], changes in sensory information [3, 48], and changes in demands of precision aiming [27, 55].

A possible transfer of postural strategy can also be supposed considering that the value of parameters as the ellipse shape or the rotation angle around the vertical axis, remained unchanged over the training time even though the amplitude of board oscillations around the horizontal plane decreased.

Further support to the idea that a common schema may be shared across diverse postural contexts is provided by the evidences that few muscle synergies may account for different perturbation directions and different levels of instability [49, 56], and that upright standing control may depend on the formation of internal models [54, 57–60]. An internal model is a set of neuronal encoded rules aimed to predict the body motion in relation to the physical environment dynamics. For example, the upright standing on a stable surface is controlled by using an internal representation of the effects of the gravitational torque on the body segments. When this type of relationship is learned in a particular context, it can be easily transferred in a novel condition by integrating the model with the variables related to the new physical environment [61]. In the case of our task, the learning process might have been accomplished by encoding the dynamics of the board movements and, by using this information, an updating of a memorized internal model was elaborated. It is possible that the rapid early improvement of the performance could partially depend on the use of these memorized structures.

#### Changes in spatio-temporal structure and frequency domain

Although the early fast phase of learning could be compatible with the functional frame discussed in the previous section, different explanations need for the changes observed for nonlinear (ApEn and FD) and frequency domain (MPF) parameters during the retention session. In fact, while stability measures maintained similar values, passing from the second to the retention session for both the directions, the structure of the temporal sway variability and the frequency content of the signal changed in the AP direction during the retention test.

Approximate entropy estimates the level of regularity of the signal in a time series, with high values indicating a more irregular motion and, therefore, a more complex response organization [37, 38]. The temporal modifications detected by this technique may depend on a more strict integration between sensory and motor elements that control upright posture. For example, proprioceptive, visual and vestibular inputs could be less associated during practice sessions, producing simple relationships between the sensory input and the motor outcome. As the linkages among the sensory channels increase, the resulting signal becomes less predictable and more complex. Similarly, the formation of new internal representation of the motor commands may be a source of complexity, since more elements are increasingly included to improve the adaptation of the motor response to the environmental requirements. Increasing of both the sensory integration and motor adaptation matches with what a postural task, such as balancing on a multiaxial board, needs to be learned. This functional schema is in line with many studies focusing on ApEn as a tool to capture the dynamics of the postural adaptations. Increase of ApEn in COP time series was observed during postconcussion recovery of postural stability [32], after training on a dynamic platform in patients with bilateral labyrinthine deficit [39], and as a consequence of the effects of secondary cognitive task on postural control [33]. In addition, ApEn values of COP were found higher in health participants compared with patients with Ehlers-Danlos syndrome [34] or Multiple Sclerosis [40]. In all of these cases, the increase of ApEn was parallel with the need to increase the complexity of the postural adaptation. Likewise, the increase of FD, also observed after the week pause, may be linked to a more complex spatial structure of the board motion.

Many authors found that the increase of ApEn or homologues parameters was focused on the AP axis [7, 32, 33, 35, 55, 62] and that this direction-dependent augmentation of complexity may depend on the level of task difficulty as suggested by Balasubramaniam et al. [55]. In fact, as the difficulty of the task increases, more joints need to be coordinated in the AP than in the ML direction, and more alternative strategies can be explored to cope with AP instability.

This line of considerations is also supported by the higher values of FD detected in the retention session with respect to the training sessions. As the FD value increases, the board trajectory gains a more complex geometrical structure. Interestingly, Duarte and Zatsiorsky, [63] reported that the level of FD observed during unconstrained upright bipedal posture, was greatly stable for long time, indicating a specific schema in the structure of static stance. The strong variation of FD observed in the current study contrasts with this stability, reinforcing the idea that novel postural structures took place during the first week of the consolidation process.

Further insights on the structure of the postural adaptations associated with the control of the multiaxial balance board may be provided by the modifications observed for the MPF. Some authors reported that changes in frequency band may be indicative of the type of sensory channel involved in the balance task: low frequencies (0–0.3 Hz) are linked to visual control, the medium-low frequency band (0.3–1 Hz) is associated with vestibular regulation, and the medium-high frequency band (>1 Hz) mostly account for proprioceptive and muscular activities [28, 29, 64]. Other studies focused on the possibility that the frequency content may be modulated by changes in the dynamic strategy of postural control [3, 30, 31].

A possible rearrangement of the coordination dynamics of the ankle, knee and hip motion to deal with AP instability may enhance the contribution of proprioceptive information [64, 65], which in turn would increase the MPF. However, the frequency increasing observed in this work (from 0.15 to 0.19 Hz) disagrees with the data reported in some studies indicating that the contribution of proprioception to balance control is associated with frequencies above 1 Hz [28, 29, 64]. On the contrary, the possibility that a new movement strategy could influence the frequency of sway oscillations reported in the current work is more in accord with the results of Fransson et al., [3], who found that fast movements are linked to frequencies above 0.1 Hz, while smooth changes in the body sway are associated with lower frequencies. Interestingly, a reduction of the body sway along AP direction was found parallel to an increase of frequency during balance improvement determined by visual biofeedback [31] or by an increase of the support instability [30]. Similarly, in this work the board sways over the AP direction moved from an initial phase, with large amplitude and low frequencies, to a final consolidation phase, with smaller oscillations and higher frequencies. Thus, we deemed that most of the contribution to the MPF increasing observed in AP direction may depend on the formation of new kinematic patterns rather than changes in sensory information.

Changes in spatio-temporal structure and frequency band showed during the retention session suggest that something more than the only amount of exercise influenced the long-term postural adaptation; that is, processes occurred during the offline periods refined and further improved the postural response. Many studies analyzing this issue found that memory consolidation occurs within a critical time frame following the practice [15], with an additional consolidation effect of the sleep [16]. Depend on the context this timing window may span from seconds [17] to several hours [15, 18], and one sleep night may be enough to influence the post-training improvements [16]. Although, these factors might have influenced the consolidation process during the week pause after the end of training, the limitation of our experimental design prevents appropriate interpretation about the role played by these factors in determining the behaviors observed in the current study.

Overall, while the comparison of amplitude and variability changes in AP and ML directions over the course of the learning process suggests that postural control evolves on the basis of previous experienced strategies, later, after the week pause, the changes in ApEn, FD and MPF may represent signals indicating the formation of new stance strategies aimed to further stabilize the learned postural performance.

### Training standing posture by using the balance board

This study provides an extensive description of the timing of adaptation and the nature of the postural processes associated with the balance board training. The rapid performance improvement, within a session of 8 trials, indicates that the effects of this type of exercise on the postural control would be already observed with a short period of training. However, an important message for applicative outcomes provided by our findings is that the possible benefits are not limited to the period of practice, but long time adaptive processes, occurring during offline periods, may be effective later in the process of functional recovery. Thus, to set an optimal paradigm for training postural abilities by using multiaxial balance boards, the rehabilitation programs would take in account that the distribution of the pauses may provide additional advantages with respect to those derived from the amount of practice alone. Future studies will be directed at the refining of the experimental design to quantify the contribution of the distribution of the practice on the upright stance stability.

Balance training on an oscillating platform is considered a specific exercise to stimulate the proprioceptive channel [66]. However, we cannot support the association between balance board training and proprioception reinforcement, since the increase of MPF in AP direction, that could be a signal for a specific proprioception contribution to the improvement of the postural performance, was not consistent with the values reported by other studies (see previous section). Generally, as the level of difficulty to maintain upright stance increase, all sensory channels significantly contribute to implement the performance [60]. In line with this idea, our data suggest that the observed benefit of training on the multiaxial balance board should depend mainly on changes in the central sensory-motor integration rather than on single sensory or motor element.

A more specific suggestions to encourage the use of multiaxial balance board for balance training, regards the anisotropic behavior observed between the AP and ML directions. In fact, the multi directional motion forced by the multiaxial board may optimize specifically this asymmetric schema of postural control with benefit for static and/or more dynamic postures which use similar modality in maintaining upright stance.

Overall, the data of the current paper may form the basis for more specific experimental designs aimed to accomplish a direct examination of people that need postural rehabilitation.

## Conclusions

Learning to maintain a stable upright stance on a multiaxial balance board stimulates a fast improvement of the performance, with long-term retention. The asymmetric stability observed between AP and ML directions suggests that a portion of the processes could have been accomplished by similar strategies experienced in other more common standing postures. In addition, changes in temporal and frequency band profiles during consolidation period indicate that the skill still develops after the practice, producing, specifically for the AP direction, new models of postural control. We deem that these findings may contribute to better understand the basic processes underlying learning and adaptation of upright posture and to help in planning a correct rehabilitation approach to the balance disorders.
